# Directional Spike Feature Learning with Progressive Reweighting for Energy-Efficient Cross-View Geo-Localization

**DOI:** 10.3390/s26175372

**Published:** 2026-08-25

**Authors:** Xin Wang, Yidan Su, Yimeng Fan, Wei Zhang, Mingyang Li

**Affiliations:** 1 Key Laboratory of Fire Protection Technology for Industry and Public Building, Ministry of Emergency Management, Tianjin 300381, China; wangxin@tfri.com.cn; 2 Tianjin Fire Science and Technology Research Institute of MEM, Tianjin 300381, China; 3 School of Microelectronics, Tianjin University, Tianjin 300072, China; suyidan2002@tju.edu.cn (Y.S.); yimengfan@tju.edu.cn (Y.F.); 4 School of Precision Instrument and Opto-Electronics Engineering, Tianjin University, Tianjin 300072, China

**Keywords:** cross-view geo-localization, spiking neural network, metric learning, hard negative mining, energy-efficient inference, UAV navigation

## Abstract

Cross-view geo-localization (CVGL) between unmanned aerial vehicle (UAV) imagery and satellite imagery is a key technique for autonomous UAV navigation in Global Navigation Satellite System (GNSS)-denied environments. However, most existing methods rely on energy-intensive Artificial Neural Networks (ANNs), making them difficult to deploy on resource-constrained edge computing platforms. Spiking Neural Networks (SNNs) provide a promising alternative for energy-efficient inference, but their application to CVGL still faces two challenges that remain insufficiently addressed. First, the isotropic computation used by existing SNN backbones is mismatched with the directional characteristics of spike activations. Spike activations tend to form oriented aggregation patterns along elongated geographic structures, and isotropic computation can therefore dilute directional signals. Second, the limited representational capacity of SNNs further increases the sensitivity during training optimization. However, the standard triplet loss adopts a static weighting strategy and assigns the same weight to all triplets that violate the margin constraint, which is unfavorable for learning from hard negatives. To address these challenges, we propose a framework with two core contributions. At the feature extraction level, the Directional Adaptive Convolution Module (DACM) processes spike feature maps by sequentially performing horizontal strip convolution and vertical strip convolution, thereby capturing a more complete geometric structure of directional spike clusters. At the training supervision level, we propose a Dual-dimensional Progressive Reweighting (DPR) loss, which jointly characterizes sample difficulty from pairwise difficulty and positive-pair quality difficulty. A learnable fusion parameter is used to adaptively balance these two types of difficulty information. Experimental results on the University-1652 and SUES-200 benchmarks show that the proposed framework, when equipped with the same representation learning head as its ANN counterparts, achieves competitive and, in many settings, superior performance. In terms of energy efficiency, its estimated theoretical energy consumption is over 8.8× lower than that of published ANN methods under their original configurations. Under a more rigorous matched ANN control that shares the identical architecture, the estimated energy is reduced from 29.84 mJ to 6.36 mJ, an approximately 4.7× reduction obtained at a cost of only 2.29 percentage points in R@1.

## 1. Introduction

Cross-view geo-localization (CVGL) has become a core capability of intelligent Unmanned Aerial Vehicle (UAV) systems operating in Global Navigation Satellite System (GNSS)-constrained environments [1,2,3]. Given a drone-view query image, CVGL retrieves the geographically corresponding image from a satellite-view gallery, thereby supporting critical applications such as autonomous UAV delivery, urban-scale navigation, and environmental monitoring [4,5,6,7,8]. Existing CVGL methods are primarily built on Artificial Neural Networks (ANNs). Representative methods, including LPN [9], TransFG [10], and Sample4Geo [11], improve retrieval accuracy through local partitioning, transformer-based feature aggregation, and hard negative sampling, respectively. However, CVGL systems are often deployed on energy-constrained UAV edge platforms, where these ANN-based methods incur substantial computational and energy costs that limit their applicability in resource-constrained settings. Spiking Neural Networks (SNNs) provide a fundamentally different computational paradigm [12,13]. By exploiting event-driven computation and sparse spike communication, SNNs can substantially reduce energy consumption compared with ANNs [14,15,16,17], making them well suited for energy-constrained CVGL deployment. Nevertheless, applying SNNs to CVGL introduces distinct representation challenges that remain largely unexplored.

Unlike the dense activations of ANNs, spike activations in SNNs are spatially sparse. Spiking neurons tend to fire in information-rich regions, forming locally concentrated spike clusters while remaining largely silent in other regions [18]. In CVGL scenes, discriminative geographic landmarks, such as roads, rivers, and buildings, often appear as elongated structures with clear spatial layouts [19,20]. Since spike responses are triggered by these salient structures, the resulting spike clusters tend to follow their spatial layouts and form directional activation patterns in the feature maps. These patterns preserve geometric cues that are important for cross-view matching. Aggregating features along the structure direction captures continuous landmark responses, whereas aggregation in the orthogonal direction mainly covers inactive or weakly informative regions. However, existing SNN backbones rely on isotropic feature extraction. Square convolutions and uniform attention mechanisms process horizontal and vertical dimensions identically, aggregating features from all directions without distinction [21]. For directional spike clusters, isotropic aggregation mixes informative activations along the landmark structure with weak or silent responses from surrounding regions. This mixing dilutes directional geographic cues and introduces redundant computation over inactive areas, thereby weakening both feature discriminability and energy efficiency. This mismatch between isotropic feature extraction and directional spike activations remains unaddressed in existing SNN-based CVGL frameworks.

Beyond the SNN-specific challenge of feature extraction, the limited representational capacity of SNNs further increases their vulnerability during training optimization. When the feature extractor cannot fully capture discriminative cues, the quality of the learned embedding becomes highly dependent on the supervision signal. However, triplet loss [22], which is the dominant objective in CVGL, assigns uniform weights to all violated triplets regardless of their difficulty. This strategy wastes learning capacity on easy samples while failing to prioritize hard negatives. Recent studies have recognized the importance of reweighting hard negatives [23], but their definitions of difficulty are inherently one-sided. They characterize hardness only by the relative proximity of the negative sample to the query, while ignoring the absolute distance between the positive sample and the query. Consequently, the actual hardness of triplets with low-quality positive samples is systematically underestimated, resulting in an insufficiently calibrated reweighting signal.

In this work, we address these challenges from two perspectives. First, to mitigate the mismatch between isotropic feature extraction and directional spike activations, we propose the Directional Adaptive Convolution Module (DACM). DACM performs sequential orthogonal strip convolutions to establish cross-directional spatial interactions, thereby capturing a more complete geometric structure of directional spike clusters and improving the discriminability of geographic features. Second, to address the one-sided difficulty definition in existing triplet-based supervision, we propose a Dual-dimensional Progressive Reweighting (DPR) loss. Sample difficulty is jointly characterized by pair-quality difficulty and pairwise difficulty. Pair-quality difficulty accounts for the absolute distance between the positive sample and the query, and pairwise difficulty measures the relative proximity of the negative sample to the query. A learnable parameter controls the relative contributions of these two dimensions. Additionally, a progressive weighting mechanism, inherited from the progressive adaptive loss weighting schedule of [23], further modulates the influence of hard negatives according to training progress, suppressing noisy gradients in early training stages and strengthening hard negative mining as training becomes stable. Extensive experiments on the University-1652 [24] and SUES-200 [25] benchmarks demonstrate consistent improvements over the corresponding baselines and accuracy competitive with state-of-the-art ANN-based methods in the evaluated settings, while preserving the energy-efficiency advantage of the SNN-based framework.

## 2. Related Work

### 2.1. Cross-View Geo-Localization

CVGL aims to determine the geographic location of a query image by matching it with geo-tagged reference images captured from different viewpoints, such as UAV, satellite, or ground-level views. It has been widely studied for UAV navigation and visual localization under GNSS-denied conditions. The University-1652 benchmark [24] extended CVGL to drone-based geo-localization, which promoted the development of UAV–satellite retrieval methods. To exploit discriminative local cues, LPN [9] proposed a square-ring partition strategy to learn part-level representations from surrounding contextual regions. Later, TransGeo [26] introduced a Transformer-based framework to capture global correlations and reduce the influence of uninformative patches. Recent studies further improve cross-view matching from different perspectives, including geometric alignment, BEV representation, orientation robustness, robustness evaluation and multimodal retrieval [27,28,29,30,31,32,33,34]. These methods significantly improve the matching accuracy and robustness of CVGL systems, but they mainly focus on enhancing feature representation and cross-view alignment within conventional ANN frameworks.

Metric learning objectives are widely used in CVGL to learn discriminative cross-view embeddings. Contrastive loss and triplet loss encourage images from the same geographic location to be close in the embedding space while pushing mismatched images apart [35]. Subsequent pair weighting and hard sample mining strategies further improve retrieval performance by emphasizing informative pairs or triplets, such as hard negatives or insufficiently optimized positive pairs [36,37]. Recent retrieval and contrastive learning studies have also shown that noisy correspondences, hard-negative ranking, and adaptive sample reweighting are important for robust representation learning [38,39,40]. However, most hard-sample strategies mainly measure difficulty from the negative side and pay limited attention to the reliability of positive cross-view pairs. In UAV–satellite geo-localization, positive pairs can also be difficult due to viewpoint changes, scale variations, occlusions, seasonal shifts, and appearance gaps. This limitation may lead to poorly calibrated supervision signals, especially when sparse spike features are more sensitive to weak or noisy geographic cues. Motivated by this issue, we propose a DPR loss that jointly considers negative-sample threat and positive-pair quality while progressively adjusting hard-sample emphasis during training.

### 2.2. Spiking Neural Networks

SNNs have attracted increasing attention as a biologically inspired computing paradigm for energy-efficient visual intelligence. Unlike conventional ANNs, SNNs transmit information through discrete spike events and update neuronal states over multiple timesteps, enabling sparse and event-driven computation. Since neurons are activated only when spike events occur, SNNs can reduce redundant multiply-and-accumulate operations and are therefore promising for low-power edge deployment [12,41]. In recent years, the development of deep visual SNNs has significantly improved their representation capability. Spikformer [42] introduced spiking self-attention to combine Transformer-style global modeling with spike-based computation. Spike-driven Transformer [43] further enforced spike-form communication and sparse addition-based attention, demonstrating the feasibility of energy-efficient Transformer-based SNNs. Following this direction, Meta-SpikeFormer [44] extended spike-driven architectures to a general visual backbone for classification, detection, and segmentation, while SpikingResformer [45] and QKFormer [46] further improved hierarchical representation learning and spiking attention design. More recent studies refine spiking attention through quantization, similarity redesign, addition-only attention, spatial–temporal modeling, and lateral inhibition mechanisms [47,48,49,50,51]. These advances indicate that SNNs are becoming competitive visual backbones for complex recognition tasks while retaining the potential advantage of low-power inference.

Despite this progress, existing visual SNN studies mainly focus on generic tasks, including image classification, object detection, semantic segmentation, and event-based perception, while CVGL remains much less explored. Directly applying existing SNN backbones to UAV–satellite geo-localization is non-trivial because CVGL depends on discriminative geographic structures, which often appear as sparse and directional spike patterns in SNN feature maps. Conventional isotropic convolution and generic spiking attention may aggregate many silent or weakly informative regions, diluting effective geographic responses and weakening cross-view discriminability. Therefore, beyond adopting an energy-efficient SNN backbone, CVGL requires task-specific feature enhancement for sparse directional spike patterns. Motivated by this observation, we propose the DACM, which progressively aggregates spike responses along horizontal and vertical directions to enhance directional geographic structures while preserving the low-power advantage of SNNs.

## 3. Method

### 3.1. Problem Statement and Method Overview

#### 3.1.1. Problem Statement

Given a query image from one view, CVGL aims to retrieve its geographically corresponding image from a gallery captured from another view. We denote the drone-view image as $x_{i}$ and the satellite-view image as $y_{j}$, where i,j∈[1,R] and *R* is the number of geographic scenes. The subscripts *i* and *j* indicate the scene indices of $x_{i}$ and $y_{j}$, respectively. When i=j, $x_{i}$ and $y_{j}$ are captured at the same geographic location. Each scene contains *N* drone-view images captured from different angles and altitudes, and one corresponding satellite-view image. CVGL involves two retrieval directions, drone-view target localization (Drone → Satellite) and drone navigation (Satellite → Drone). The former retrieves the corresponding satellite-view image using a drone-view query, while the latter retrieves matching drone-view images using a satellite-view query.

Formally, given a query image $x_{i}$ and a gallery set $\left\{y_{1},y_{2},\ldots ,y_{R}\right\}$, the goal is to find the gallery image $y_{i}$ that best matches $x_{i}$ in a learned feature space. A weight-sharing dual-branch encoder F(·) is adopted to project images from both views into a shared embedding space, such that images of the same geographic location are close while images of different locations are far apart. The retrieval objective is formulated as:

(1) $$y_{i}^{*}=\underset{y_{j}}{\arg\min}\;D\;\left(F\left(x_{i}\right),\;F\left(y_{j}\right)\right)$$

where D(·,·) denotes the Euclidean distance between two embeddings. All embeddings are $\ell_{2}$-normalized before matching. For unit-norm vectors the squared Euclidean distance and the cosine distance satisfy ${∥a-b∥}_{2}^{2}=2\;\left(1-\cos\left(a,b\right)\right)$, a strictly monotonic relation, so ranking by Euclidean distance, by squared Euclidean distance and by cosine distance is identical. Consequently the retrieval objective in Equation (1), the squared-Euclidean triplet objective used for training, and the cosine-distance retrieval reported in Section 4.2 are rank-equivalent and are used interchangeably throughout this paper.

#### 3.1.2. Method Overview

**Figure 1:**
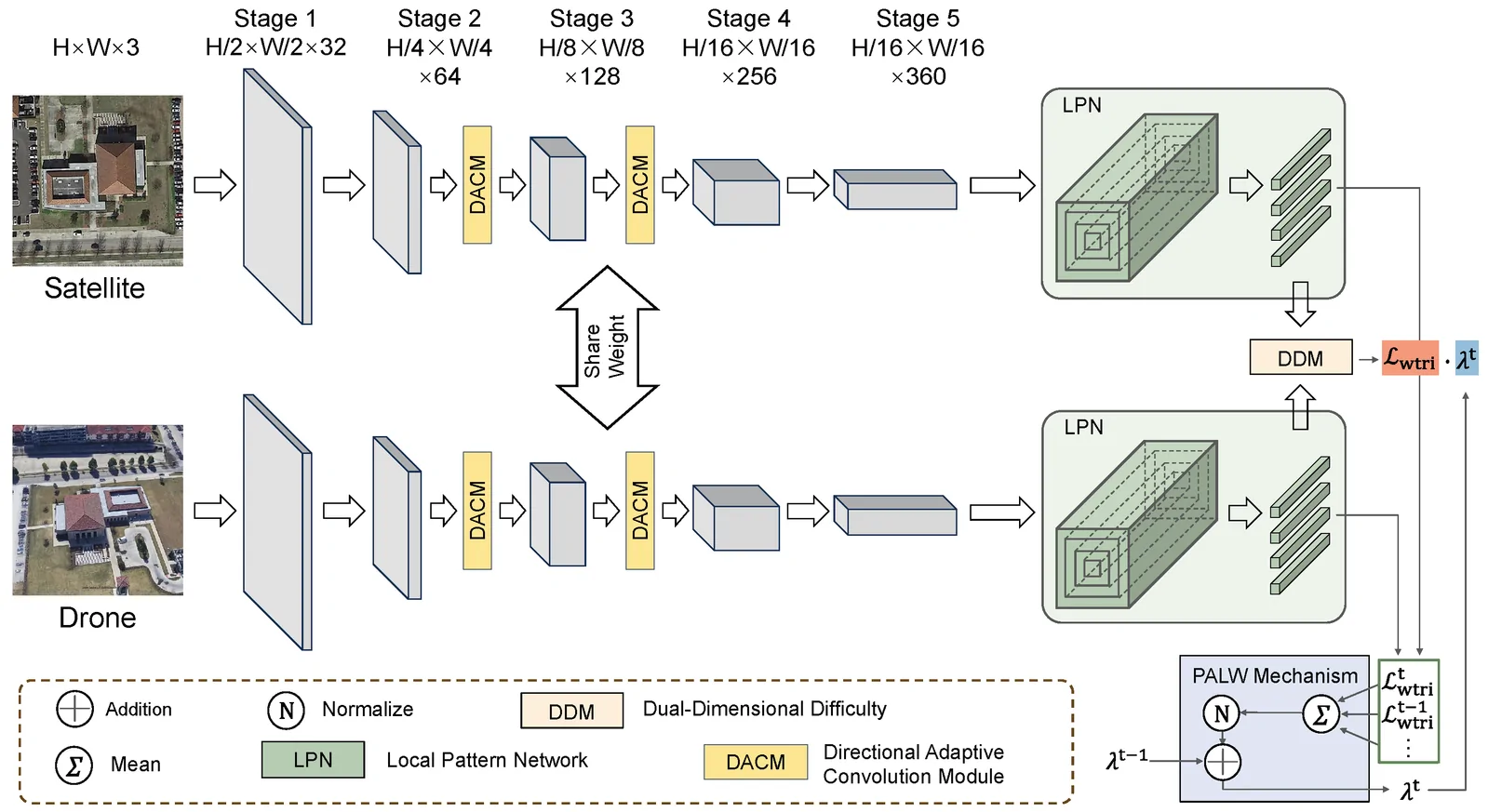
The overview of our methods.

As illustrated in Figure 1, the proposed framework follows a dual-branch weight-sharing encoder architecture consisting of three sequential components. The backbone network adopts E-SpikeFormer [52], a spike-driven transformer that achieves energy-efficient feature extraction through event-driven sparse computation. To address the misalignment between isotropic feature extraction and the directional nature of spike activations in CVGL scenes, we insert the proposed DACM at the shallow stages of the backbone, where spike activations retain strong directional characteristics. DACM enriches the extracted features with directional geometric information. Following the backbone, the feature maps are then partitioned into concentric square-ring regions according to LPN [9], capturing hierarchical contextual representations from the geographic target outward. Each partitioned region is subsequently processed by a shared classifier module to generate a descriptor. During inference, all part-level descriptors are concatenated to form the final image embedding, and retrieval is performed via nearest-neighbor search in the embedding space.

The overall training objective combines the cross-entropy loss, the standard triplet loss [22] and the proposed DPR loss. It is formulated as:

(2) $$L=L_{\mathrm{CE}}+L_{\mathrm{tri}}+L_{\mathrm{DPR}}$$

where $L_{\mathrm{CE}}$ denotes the cross-entropy classification loss applied to each part-level descriptor, $L_{\mathrm{tri}}$ provides the standard triplet loss, and $L_{\mathrm{DPR}}$ denotes the proposed DPR loss. The detailed formulation of $L_{\mathrm{DPR}}$ is introduced in Section 3.3. We emphasize that Equation (2) is the objective used in all experiments reported in this paper: $L_{\mathrm{DPR}}$ is an additional, progressively weighted term rather than a replacement of $L_{\mathrm{tri}}$, and the standard triplet loss is retained in every configuration. Retaining $L_{\mathrm{tri}}$ is deliberate: because $\lambda^{t}$ starts at $\delta_{\min}=0$, an objective containing only the reweighted term would provide almost no metric-learning signal during the early epochs. A control experiment that excludes the alternative explanation that the observed gain merely reflects a larger total weight on metric-learning supervision is reported in Table 7.

For each query embedding $q_{i}$ in a mini-batch, we construct triplets $\left(q_{i},p_{i},n_{i,k}\right)$, where $p_{i}$ is its corresponding positive embedding and $n_{i,k}$ is the *k*-th negative embedding. The standard triplet loss is defined as:

(3) $$L_{\mathrm{tri}}=\frac{1}{B(B-1)}\sum_{i=1}^{B}\sum_{k=1}^{B-1}\ell_{\mathrm{tri}}\left(i,k\right),$$

where $\ell_{\mathrm{tri}}\left(i,k\right)=\max\left(0,\;d\left(q_{i},p_{i}\right)-d\left(q_{i},n_{i,k}\right)+m\right)$, and m>0 denoting the margin hyperparameter. Here, $d\left(a,b\right)={∥a-b∥}_{2}^{2}$ denotes the squared Euclidean distance.

### 3.2. Directional Adaptive Convolution Module (DACM)

In CVGL scenes, directional geographic structures frequently induce oriented spike clusters in SNN feature maps, a tendency that we quantify in Section 4.4.1. However, existing SNN backbones rely on isotropic square convolutions, which aggregate information uniformly across both spatial dimensions and thus weaken feature discriminability [21]. To address this mismatch, we propose DACM, which replaces isotropic aggregation with a sequential orthogonal strip-convolution design.

**Figure 2:**
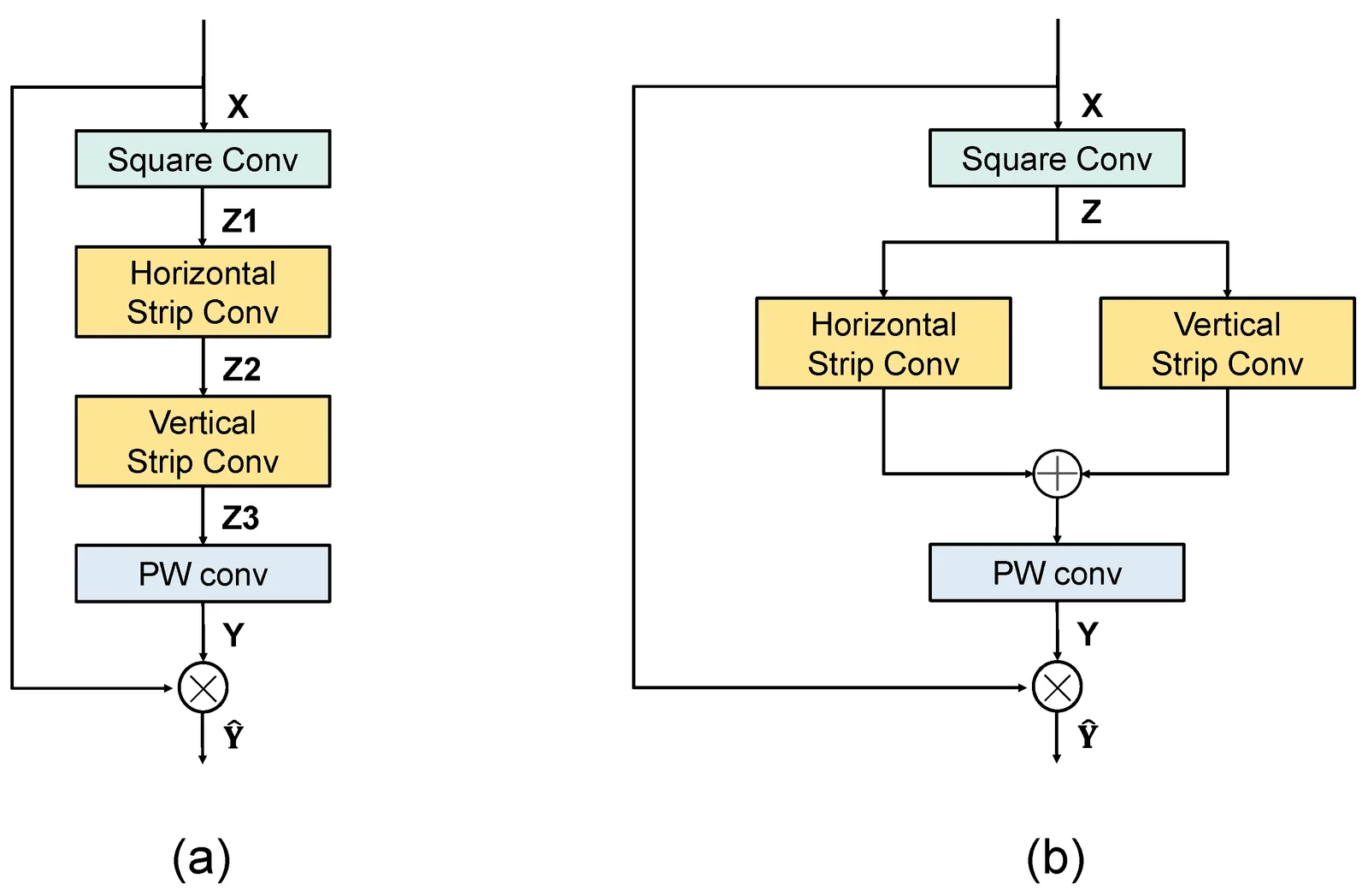
Structure of DACM. (**a**) The adopted sequential design, in which the vertical strip convolution operates on horizontally aggregated features to establish cross-directional spatial interaction. (**b**) A parallel alternative, in which horizontal and vertical strip convolutions process the same input independently and their outputs are added.

Given an input feature map $X\in R^{C\times H\times W}$, DACM enhances directional spike responses through a lightweight sequential convolutional design, as illustrated in Figure 2a. It first applies a depthwise square convolution with a kernel size of $k_{s}\times k_{s}$ (set to 5×5) to capture the local spatial context of individual spike clusters, where $k_{s}$ is set to 5 in our implementation. Based on the locally enriched features, a depthwise horizontal strip convolution with a $1\times k_{h}$ kernel is then used to aggregate spike activations over an extended horizontal neighborhood. Subsequently, a depthwise vertical strip convolution with a $k_{v}\times 1$ kernel further aggregates the horizontally enhanced features along the vertical direction. Through this sequential horizontal-to-vertical aggregation, DACM enables each spatial position to encode directional responses from a broad two-dimensional receptive field, which is beneficial for modeling elongated geographic structures such as roads, rivers, and building contours. The specific values of $k_{h}$ and $k_{v}$ are determined by the ablation experiments in Section 4.4. Finally, a pointwise convolution is applied to fuse channel-wise information and generate a reweighting map $Y\in R^{C\times H\times W}$. This map is multiplied with the original input feature map to obtain the final output $\hat{Y}=X⊙Y$, thereby suppressing weak responses from inactive regions while highlighting directional geographic cues captured by the strip convolutions. For notational convenience, we denote by $Z_{1}$, $Z_{2}$ and $Z_{3}$ the outputs of the depthwise square, horizontal strip and vertical strip convolutions, respectively.

It is worth noting that the sequential ordering of the horizontal and vertical strip convolutions is crucial. Specifically, the vertical strip convolution operates on the horizontally aggregated feature map $Z_{2}$, rather than directly on the original input. Consequently, each spatial position in $Z_{3}$ encodes information gathered from a broad two-dimensional neighborhood, establishing cross-directional spatial interaction. In contrast, the parallel variant independently applies horizontal and vertical strip convolutions to the same input and sums their outputs. This design cannot effectively capture the coupled two-dimensional geometry of directional spike clusters because the two directions are processed without mutual conditioning. This difference can be made precise in terms of receptive-field composition. In the parallel variant the effective support of the strip pair is the union of a $1\times k_{h}$ row and a $k_{v}\times 1$ column centered at the same location, i.e., a cross-shaped region containing $k_{h}+k_{v}-1$ positions; because the two branches read the same input and are merely summed, the response is an additive combination of two one-dimensional statistics and no term depends jointly on a pair of offsets (Δx,Δy) with Δx≠0 and Δy≠0. In the sequential design the vertical kernel is applied to an already horizontally aggregated map, so the composed operator is a separable filter with full rectangular support of $k_{h}\times k_{v}$ positions whose weights factorize as $w_{v}\left(\Delta y\right)\;w_{h}\left(\Delta x\right)$, and the response therefore depends on both offsets simultaneously. For the adopted setting $k_{h}=k_{v}=17$, this corresponds to 289 positions against 33 for the parallel variant, an 8.8× larger effective support at essentially identical parameter cost. Oblique and curved structures, whose support is aligned with neither axis, are consequently modeled substantially more effectively by the sequential composition, which provides a much broader joint two-dimensional support for such off-axis patterns, which is consistent with the empirical gap reported in Table 6.

We insert DACM into stages 2 and 3 of the E-SpikeFormer backbone, based on the spatial characteristics of spike activations across network depths. In the shallow stages, spike activations retain strong directional structure corresponding to low-level geographic patterns such as edges and contours, where the large-span strip convolutions can aggregate meaningful directional signal. In the deep stages, spike activations become increasingly sparse and spatially dispersed due to cumulative quantization errors and progressive feature abstraction, such that large-span strip convolutions would aggregate predominantly silent regions and degrade feature discriminability.

### 3.3. Dual-Dimensional Progressive Reweighting (DPR) Loss

The standard triplet loss (Equation (3)) assigns equal gradient contributions to all violated triplets regardless of their relative difficulty, and applies a static weighting strategy that prematurely emphasizes hard negatives before embeddings are sufficiently structured. Recent reweighting approaches [23] partially address these issues, yet their difficulty definition remains one-sided, characterizing hardness solely through the relative proximity of the negative to the query while ignoring the quality of the positive sample. We propose a dual-dimensional difficulty definition that accounts for both sources of hardness, yielding a more complete and better-calibrated reweighting signal.

The first dimension, pairwise difficulty, measures how threatening the negative is relative to the positive:

(4) $$h_{\mathrm{pair},i,k}=\frac{d(p_{i},q_{i})}{d\left(p_{i},q_{i}\right)+d\left(q_{i},n_{i,k}\right)}.$$

A larger $h_{\mathrm{pair},i,k}$ indicates that the negative is closer to the query relative to the positive, corresponding to a more confusing triplet. This ratio-based formulation is scale-invariant under global distance rescaling, avoiding weight drift caused by varying feature norms across training stages.

The second dimension, pair quality difficulty, captures the reliability of the positive sample as a supervision signal:

(5) $$h_{\mathrm{quality},i}=\frac{d(p_{i},q_{i})}{{\bar{d}}_{\mathrm{pos}}+\epsilon }$$

where ${\bar{d}}_{\mathrm{pos}}=\frac{1}{B}\sum_{i=1}^{B}d\left(p_{i},q_{i}\right)$ is the batch-wise mean positive distance serving as a normalization baseline, and ϵ is a small constant for numerical stability. A larger $h_{\mathrm{quality},i}$ indicates that the positive sample is far from the query relative to the batch average, reflecting a globally difficult triplet. Note that $h_{\mathrm{quality},i}$ is shared across all negatives *k* for the same anchor *i*, as it characterizes a property of the positive pair rather than of any individual negative.

Because $h_{\mathrm{pair},i,k}\in \left(0,1\right)$ by construction whereas $h_{\mathrm{quality},i}$ is not bounded from above, the latter is saturated before fusion so that the two dimensions share a common scale:

(6) $${\hat{h}}_{\mathrm{quality},i}=\min\left(\frac{h_{\mathrm{quality},i}}{\tau },\;1\right),$$

where τ is a saturation threshold set to τ=2, so that a positive distance twice the batch mean already corresponds to maximal pair-quality difficulty. Besides placing the two dimensions on the same scale, this saturation bounds the influence of a single outlying positive pair, as discussed below.

**Figure 3:**
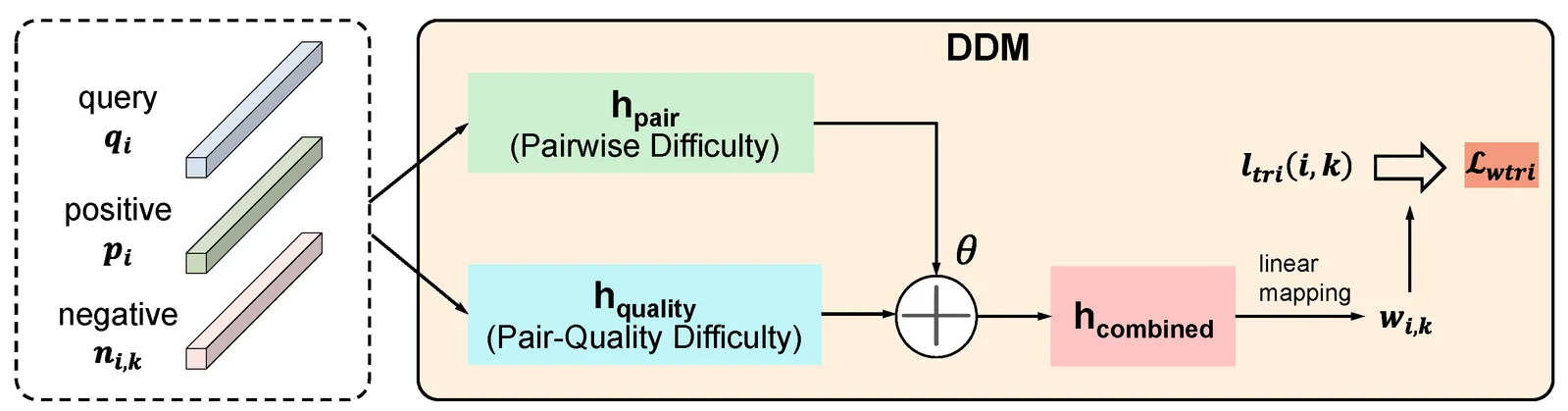
Overview of the dual-dimensional difficulty modeling (DDM).

As illustrated in Figure 3, the two difficulty dimensions are combined into a unified score via a learnable weighted fusion:

(7) $$h_{\mathrm{combined},i,k}=\sigma \left(\theta \right)\cdot h_{\mathrm{pair},i,k}+\left(1-\sigma (\theta )\right)\cdot {\hat{h}}_{\mathrm{quality},i},$$

where $\sigma \left(\theta \right)=1/\left(1+e^{-\theta }\right)$ is the sigmoid function applied to a learnable scalar parameter θ, ensuring the fusion weight remains in (0,1). Unlike a fixed hyperparameter, the learnable θ allows the model to automatically determine the optimal balance between the two difficulty dimensions during training, adapting to the specific characteristics of the training distribution without manual tuning. Since both terms of Equation (7) lie in [0,1], so does $h_{\mathrm{combined},i,k}$. The combined hardness score is mapped to a sample weight by the fixed affine transform:

(8) $$\omega_{i,k}=w_{\min}+\left(w_{\max}-w_{\min}\right)\;h_{\mathrm{combined},i,k},$$

so that $\omega_{i,k}\in \left[w_{\min},w_{\max}\right]$ holds exactly for every triplet and no clipping, batch-wise renormalization or additional rescaling of $\omega_{i,k}$ is required beyond the batch-mean normalization already contained in Equation (5) and the saturation of Equation (6). All distances entering Equations (4) and (5) are detached from the computational graph, i.e., the difficulty scores are treated as constants during backpropagation. Consequently, no gradient flows to the embedding through $\omega_{i,k}$, which acts purely as a per-triplet modulation of the gradient magnitude; this prevents the network from reducing the loss by manipulating its own difficulty estimates instead of improving the embedding. The single exception is the fusion parameter θ, which receives gradients through σ(θ) in Equation (7) and is optimized jointly with the network. With these definitions, the hardness-weighted triplet loss is formulated as:

(9) $$L_{\mathrm{wtri}}=\frac{1}{B(B-1)}\sum_{i=1}^{B}\sum_{k=1}^{B-1}\omega_{i,k}\cdot \ell_{\mathrm{tri}}\left(i,k\right).$$

To prevent noisy gradients from destabilizing early-stage optimization, we adopt the Progressive Adaptive Loss Weighting (PALW) mechanism [23], which dynamically regulates the influence of $L_{\mathrm{wtri}}$ based on a training-progress signal. Specifically, the proposed DPR loss is defined as:

(10) $$L_{\mathrm{DPR}}=\lambda^{t}L_{\mathrm{wtri}},$$

where $\lambda^{t}$ controls the strength of hardness-aware reweighting at training iteration *t*. As shown in Figure 1, the progress signal is derived from the unweighted triplet loss. Let $L_{\mathrm{tri}}^{\left(j\right)}$ denote its value at iteration *j*. A sliding-window average over the most recent $R_{t}$ iterations is computed as

(11) $$\alpha^{t}=\frac{1}{R_{t}}\sum_{j=t-R_{t}+1}^{t}L_{\mathrm{tri}}^{\left(j\right)},$$

and normalized by the running extrema $\alpha_{\min}^{t}$ and $\alpha_{\max}^{t}$ observed up to iteration *t*,

(12) $${\hat{\alpha }}^{t}=\mathrm{clip}\;\left(\frac{\alpha^{t}-\alpha_{\min}^{t}}{\alpha_{\max}^{t}-\alpha_{\min}^{t}+\epsilon },\;0,\;1\right).$$

The instantaneous scaling coefficient is then

(13) $${\tilde{\lambda }}^{t}=\delta_{\min}+\left(\delta_{\max}-\delta_{\min}\right)\left(1-{\hat{\alpha }}^{t}\right),$$

and is smoothed by an exponential moving average to avoid abrupt changes of the effective objective,

(14) $$\lambda^{t}=\beta \;\lambda^{t-1}+\left(1-\beta \right)\;{\tilde{\lambda }}^{t},\;\lambda^{0}=\delta_{\min}.$$

We use $R_{t}=50$ iterations, β=0.9, $\epsilon =10^{-8}$ and $\left[\delta_{\min},\delta_{\max}\right]=\left[0.0,1.0\right]$. Because $\alpha^{t}$ is large early in training, ${\hat{\alpha }}^{t}$ is close to one and $\lambda^{t}$ remains near $\delta_{\min}$, suppressing the influence of hard-negative reweighting; as the embedding stabilizes and the unweighted triplet loss decreases, ${\hat{\alpha }}^{t}$ decreases and $\lambda^{t}$ approaches $\delta_{\max}$, progressively strengthening hard-negative mining.

The running extrema are initialized at the first optimization step, at which the sliding window contains a single value, by setting $\alpha_{\min}^{1}=\alpha_{\max}^{1}=\alpha^{1}$; thereafter they are updated online as $\alpha_{\min}^{t}=\min_{j\leq t}\alpha^{j}$ and $\alpha_{\max}^{t}=\max_{j\leq t}\alpha^{j}$. To resolve the degeneracy of Equation (12) while the two extrema still coincide, we define ${\hat{\alpha }}^{t}=1$ whenever $\alpha_{\max}^{t}-\alpha_{\min}^{t}<\epsilon$, so that ${\tilde{\lambda }}^{t}=\delta_{\min}$ and hardness-aware reweighting remains inactive during this brief warm-up phase. Reweighting is therefore activated only once the observed progress signal has spread enough to define a meaningful normalization range, which is consistent with the intended schedule in which $\lambda^{t}$ departs from $\delta_{\min}$ only as training stabilizes.

For clarity we state explicitly which elements of the proposed loss are inherited and which are new. The progressive weighting schedule of Equations (11)–(14), including the use of a training-progress signal and its exponential smoothing, is adopted unchanged from PALW [23], and the pairwise difficulty of Equation (4) also originates from that work. The contributions of the present paper at the supervision level are: (i) the pair-quality difficulty of Equations (5) and (6), which introduces an absolute, batch-referenced notion of triplet hardness that is absent from PALW; (ii) the dual-dimensional fusion of Equation (7) together with the learnable parameter θ, which removes the need to balance the two difficulty notions by hand; and (iii) the integration of this formulation into a spike-driven CVGL pipeline. The empirical decomposition of these elements is reported in Table 7.

The two difficulty dimensions are designed to be complementary, but they are not statistically independent: both Equations (4) and (5) contain the positive distance $d(p_{i},q_{i})$, so a positive correlation is expected by construction. We therefore measured the Pearson correlation between $h_{\mathrm{pair},i,k}$ and ${\hat{h}}_{\mathrm{quality},i}$ over all triplets of 500 randomly sampled training batches at three stages of optimization. It is 0.41 at epoch 1, 0.46 at epoch 60 and 0.44 at epoch 120, which is moderate and stable rather than negligible. We accordingly describe the two dimensions as complementary but partially correlated: each explains a fraction of the variance of triplet hardness that the other does not, which is exactly what the ablation of Table 7 measures, and we avoid any claim of orthogonality or statistical independence.

A large positive distance does not necessarily indicate an informative hard positive; it may equally reflect an annotation error, an outlying viewpoint, or extreme appearance variation, so pair-quality reweighting could in principle amplify noisy positive pairs. Three mechanisms bound this risk. First, the saturation of Equation (6) caps the contribution of any positive pair whose distance exceeds τ times the batch mean, so a single outlier cannot dominate the fused score. Second, the affine map of Equation (8) restricts the weight to $\left[w_{\min},w_{\max}\right]=\left[0.5,1.5\right]$, so the ratio between the largest and the smallest attainable weight is only three, in contrast to exponential or self-paced schemes whose weights are unbounded. Third, the progressive coefficient $\lambda^{t}$ suppresses the entire reweighted term during the early epochs, when the embedding is least reliable and mislabeled pairs are hardest to distinguish from genuinely difficult ones. No explicit noise-filtering procedure is applied beyond these mechanisms, and robust positive-pair selection remains a natural direction for future work.

## 4. Experiments

### 4.1. Datasets and Evaluation Metrics

#### 4.1.1. Datasets

**University-1652** [24] is a large-scale multi-source benchmark for CVGL, comprising images of 1652 buildings from 72 universities worldwide. Each building is associated with one satellite-view image and approximately 54 drone-view images simulated from Google Earth 3D models at varying altitudes and orientations, yielding roughly 50,000 training images in total. The dataset is partitioned by building identity into disjoint training and test splits: the training set covers 701 buildings from 33 universities, while the test set covers the remaining 951 buildings from 39 universities. Two retrieval tasks are defined on the test split. For drone-view target localization (Drone → Satellite), each drone-view image serves as a query against a satellite-view gallery augmented with 250 distractors. For drone navigation (Satellite → Drone), each satellite-view image is used as a query against a drone-view gallery containing 13,500 distractors. The large-scale distractor pools and substantial viewpoint discrepancies make University-1652 a challenging and widely adopted benchmark for evaluating cross-view retrieval robustness.

**SUES-200** [25] is a real-drone CVGL dataset collected across diverse scene types, including parks, campuses, lakes, and urban infrastructure. It contains 200 geographic scenes split into 120 for training and 80 for testing. For each scene, drone-view images are captured at four fixed altitudes of 150 m, 200 m, 250 m, and 300 m, with 50 images per altitude, alongside one satellite-view image per scene. The test gallery incorporates samples from both training and test splits as additional distractors, increasing retrieval difficulty. The multi-altitude design makes SUES-200 particularly suitable for evaluating robustness to scale variation and altitude-induced appearance change.

#### 4.1.2. Evaluation Metrics

We evaluate retrieval performance using **Recall@1** (R@1) and **Average Precision** (AP), which are standard metrics in the CVGL literature [9,24]. R@1 measures the proportion of queries for which the correct match appears as the top-ranked retrieval result, reflecting the precision of the highest-confidence predictions. AP evaluates the overall quality of the ranked retrieval list by computing the area under the precision–recall curve for each query and averaging over all queries. The two metrics are complementary: R@1 captures top-1 accuracy, while AP is sensitive to the complete ranking and is particularly informative when a scene has multiple correct matches, as in the Satellite → Drone task of University-1652.

To assess energy efficiency, we adopt the theoretical hardware-agnostic energy estimation protocol widely used in the SNN literature [16,43,53,54]. Under the 45 nm CMOS technology node, the energy cost of a multiply–accumulate (MAC) operation is $E_{\mathrm{MAC}}=4.6$ pJ and that of an addition (AC) operation is $E_{\mathrm{AC}}=0.9$ pJ. For ANN-based methods, total energy is estimated as:

(15) $$E_{\mathrm{ANN}}=\mathrm{FLOPs}\times E_{\mathrm{MAC}}/2.$$

For our SNN-based framework, energy consumption is decomposed into two components. Non-spiking layers (the patch embedding and classification head) contribute:

(16) $$E_{\mathrm{non}-\mathrm{spike}}={\mathrm{FLOPs}}_{\mathrm{non}-\mathrm{spike}}\times E_{\mathrm{MAC}}/2.$$

The spiking backbone consumes:

(17) $$E_{\mathrm{spike}}=\mathrm{SOPs}\times E_{\mathrm{AC}},$$

where synaptic operations (SOPs) are computed as:

(18) $$\mathrm{SOPs}=\sum_{t=1}^{T}\sum_{l=1}^{L}{\mathrm{FLOPs}}^{\left(l\right)}\times r_{t}^{\left(l\right)},$$

with *T* denoting the number of timesteps, *L* the number of spiking layers, and $r_{t}^{\left(l\right)}$ the measured firing rate of layer *l* at timestep *t*. The total energy consumption of the SNN is then:

(19) $$E_{\mathrm{SNN}}=E_{\mathrm{non}-\mathrm{spike}}+E_{\mathrm{spike}}.$$

This estimation framework enables a consistent first-order comparison of energy efficiency between SNN and ANN architectures.

Because energy efficiency is one of the central claims of this work, we state the assumptions and limitations of this protocol here rather than deferring them to the conclusion. The protocol (i) counts arithmetic operations only and ignores memory access, on-chip and off-chip data movement, control logic and static leakage, which often dominate the measured power of real accelerators; (ii) assumes a single 45 nm technology node for both SNN and ANN, so the reported values are technology-normalized rather than device-specific; (iii) charges one AC operation per synaptic operation in spike-driven layers, which presupposes an event-driven substrate that actually skips zero activations, a condition met by neuromorphic processors but not by GPUs; and (iv) treats the non-spiking patch embedding and head as dense MAC computation. The reported numbers should therefore be read as a hardware-agnostic estimate of arithmetic energy and as a relative rather than an absolute measure. We nevertheless adopt it because it is the protocol used by the SNN works we compare against [16,43,53,54], which makes our numbers directly comparable with the published literature. Measured energy and latency on neuromorphic hardware are discussed as future work in the Conclusions.

The firing rates $r_{t}^{\left(l\right)}$ in Equation (18) are not free parameters but are measured empirically. For each dataset we run inference over the complete test query set with the trained model, accumulate for every spiking layer the number of emitted spikes and the number of neurons, and obtain $r_{t}^{\left(l\right)}$ as the ratio of the two, averaged over all test samples and over the T×D firing steps of the Spike Firing Approximation neuron. SOPs are then obtained by weighting the layer-wise FLOPs by these measured rates and summing over layers, and the reported energy is the corresponding test-set average. Because $r_{t}^{\left(l\right)}$ depends on the input statistics, the same architecture yields slightly different SOP counts on different datasets; this is precisely why the estimated energy of an identical model is 6.36–6.41 mJ on University-1652 and 7.63–7.69 mJ on SUES-200. The higher value on SUES-200 reflects the larger proportion of textured natural scenes such as parks, lakes and vegetation in that benchmark, which elicit denser spiking than the comparatively homogeneous campus imagery of University-1652. Representative firing-rate statistics are reported in Section 4.4.1.

The cost of DACM is fully included in this accounting. The depthwise square, horizontal strip and vertical strip convolutions receive spike-form inputs and are therefore counted as accumulate operations, with their FLOPs weighted by the measured firing rate of the preceding spiking neuron layer. The pointwise convolution that produces the reweighting map Y operates on the output of the strip-convolution chain, which is no longer binary; together with the element-wise product $\hat{Y}=X⊙Y$ it is conservatively counted as dense multiply–accumulate computation and included in $E_{\mathrm{non}-\mathrm{spike}}$. DACM thus does introduce dense-valued intermediate tensors inside the module, and we do not claim that it is spike-driven end to end; the spike form is restored at the module output by the spiking neuron of the following block, so that all subsequent backbone stages remain spike-driven. Quantitatively, the two DACM instances account for 0.42 mJ, that is 6.6% of the total estimated inference energy, and for 0.34 M parameters.

### 4.2. Implementation Details

All experiments are conducted on a single NVIDIA RTX 8000 GPU. The backbone network adopts E-SpikeFormer [52] with Spike Firing Approximation configured as T×D=1×4, where *T* denotes the number of timesteps and *D* denotes the firing bound. Input images are resized to 384×384. Data augmentation includes random cropping, random horizontal flipping, color jittering, and multi-sampling.

The network is trained for 120 epochs using SGD with momentum 0.9 and weight decay $5\times 10^{-4}$. The batch size is set to 8. The initial learning rate is set to 0.01 and decays following a cosine annealing schedule. The DACM is inserted at stages 2 and 3 of the backbone, with the square convolution kernel size $k_{s}=5$ and the strip convolution kernel sizes $k_{h}=k_{v}=17$, as determined by the ablation studies in Section 4.4. The square-ring partition module follows the LPN [9] strategy with K=4 concentric rings.

For the DPR loss, the sample weight range is set to $\left[w_{\min},w_{\max}\right]=\left[0.5,1.5\right]$, and the scaling coefficient $\lambda^{t}$ in Equation (2) is bounded by $\left[\delta_{\min},\delta_{\max}\right]=\left[0.0,1.0\right]$ following the progressive schedule of the prior work [23]. The learnable fusion parameter θ is initialized to 0 (i.e., σ(θ)=0.5, giving equal initial weight to both difficulty dimensions) and is optimized jointly with the rest of the network. The triplet loss margin is set to m=0.3.

For reproducibility we further specify the following settings. The backbone is initialized from the ImageNet-1k pretrained E-SpikeFormer-L checkpoint released by the authors of [52], while DACM and all part-level classifiers are trained from scratch. The spiking neuron is the integer leaky integrate-and-fire neuron used by the Spike Firing Approximation of E-SpikeFormer with T×D=1×4, a membrane decay constant of 0.25, a firing threshold of 1.00 and hard reset to the resting potential after firing; the rectangular surrogate gradient with width 1.00 is used in the backward pass. Each mini-batch is composed of eight geographic identities, each contributing one satellite-view and one drone-view image, so that every anchor has exactly one within-batch positive and B−1=7 within-batch negatives; all valid triplets of the batch are used rather than a mined subset, consistent with the double summation in Equation (3). The final descriptor is the concatenation of the K=4 part-level features of dimension 512 each, giving a 2048-dimensional representation, and retrieval uses the cosine distance. All experiments use random seeds {0,1,2} with deterministic cuDNN kernels; unless a mean and a standard deviation are given, the value obtained with seed 0 is reported.

Two remarks on the DPR loss are in order. First, $h_{\mathrm{quality}}$ is normalized by the batch-wise mean positive distance while the batch size is only eight, so ${\bar{d}}_{\mathrm{pos}}$ is a noisy estimator whose standard error scales as $O(B^{-1/2})$. Three factors limit the practical impact of this noise: the saturation of Equation (6), the bounded weight range [0.5,1.5], and the exponential smoothing of $\lambda^{t}$, which averages the effective supervision strength over many iterations. Retraining the full model with a batch size of 16, changes Drone → Satellite R@1 by 0.21 percentage points, indicating that the method is not critically sensitive to this choice within the range that fits on a single GPU at 384×384 resolution. Second, the learnable fusion parameter converges to σ(θ)= 0.63 at the end of training, so the model assigns the larger share of the fused difficulty score to the pairwise dimension while retaining a substantial contribution from pair quality. This is consistent with Table 7, in which $h_{\mathrm{pair}}$ alone outperforms $h_{\mathrm{quality}}$ alone yet neither matches their combination.

### 4.3. Comparison with State-of-the-Art Methods

We evaluate our method on both the University-1652 and SUES-200 benchmarks. Since the primary contribution of this work targets the backbone level, we design the comparison to isolate the effect of these contributions from the choice of part-based representation learning head. Specifically, we fix the SNN backbone as E-SpikeFormer-L equipped with DACM and trained with the proposed loss, and systematically vary the part-based representation learning head by substituting it with the head structures of four representative published methods: LPN [9], FSRA [55], SDPL [56], and Safe-Net [57]. In each configuration, only the head structure is borrowed; all backbone components remain unchanged. These configurations are denoted as “Ours + [Head]”. This design allows a controlled assessment of whether the proposed backbone-level improvements are head-agnostic and generalize across different representation learning strategies.

A consistent and substantial advantage in computational efficiency is observed across all our configurations. Compared to the ANN-based methods, which consume between 56.21 mJ and 225.52 mJ, our SNN-based configurations require only 6.36–6.41 mJ on University-1652 and 7.63–7.69 mJ on SUES-200, representing a reduction of approximately one order of magnitude. This efficiency advantage is preserved regardless of the choice of representation learning head, since the dominant energy cost originates from the spike-driven backbone rather than the head. Similarly, our configurations use at most 23.02 M parameters, substantially fewer than most ANN-based counterparts. These results confirm that the proposed framework achieves competitive retrieval accuracy while maintaining the energy efficiency intrinsic to SNN-based inference.

Two aspects of Table 1 and Table 2 require care in interpretation. First, the compared methods operate at different input resolutions: the ANN baselines are reported under their original configurations (256×256 or 512×512), whereas our configurations use 384×384. Resolution affects accuracy and energy jointly and in the same direction, since the operation count of a convolutional or transformer backbone grows approximately quadratically with the side length. Our setting is therefore intermediate: relative to the 512×512 baselines we process fewer pixels and are favored in the energy column, whereas relative to the 256×256 baselines we process more pixels and are penalized in the energy column while being potentially favored in accuracy. Because these two groups bracket our configuration and the observed energy gap exceeds one order of magnitude with respect to both, the efficiency conclusion is not an artifact of the resolution mismatch. Second, and more fundamentally, the ANN entries also differ in backbone, training schedule and loss, so they characterize the published state of the art rather than a controlled comparison.

To isolate the effect of spike-driven computation from these confounding factors, we additionally train a matched ANN control. It is obtained from the proposed model by replacing every spiking neuron with a ReLU activation and removing the temporal dimension, while keeping the depth, width, embedding dimensions, patch embedding, DACM configuration, LPN head, objective (Equation (2)), input resolution, optimizer, learning-rate schedule, augmentation and number of epochs strictly identical; its energy is estimated with Equation (15), as for the other ANN entries. This control differs from our model in exactly one respect, namely the activation function and the associated event-driven computation, and is therefore the reference against which the accuracy–energy trade-off should be read. As reported in the last block of Table 1, the ANN control attains 84.86% R@1 on Drone → Satellite at an estimated 29.84 mJ, against 82.57% R@1 at 6.36 mJ for the spiking model; that is a difference of 2.29 percentage points in accuracy for a 4.7× reduction in estimated energy. This quantifies the price of spike-driven computation under otherwise identical conditions and, in our view, characterizes the trade-off more meaningfully than a comparison with heterogeneous published baselines.

**Table 1:** Performance comparison on University-1652. The upper section reports ANN-based methods under their original configurations. The lower section reports our SNN-based configurations, in which the backbone is fixed as E-SpikeFormer-L equipped with DACM and trained with the proposed DPR loss, while only the part-based representation learning head is replaced with that of the indicated method. “Ours + [Head]” denotes that only the head structure is borrowed; all other components remain unchanged. Energy is estimated following the hardware-agnostic protocol described in Section 4.1.2. The last block reports a matched ANN control obtained by replacing every spiking neuron of the proposed model with a ReLU activation, all other settings being identical. The best performance within each section is marked in bold.

Method	Publication	Image Size	Backbone	Param	Energy	Drone → Satellite		Satellite → Drone	
				(M)	(mJ)	R@1	AP	R@1	AP
*ANN-based methods*									
Contrastive Loss	CVPR’15	512×512	ResNet-50	-	-	52.39	57.44	63.91	52.24
Soft Margin Triplet	PR’18	512×512	ResNet-50	-	-	53.21	58.03	65.62	54.47
Triplet	-	512×512	ResNet-50	-	-	55.18	59.97	63.62	53.85
LPN	TCSVT’22	512×512	ResNet-50	62.39	84.59	75.93	79.14	86.45	74.79
RK-Net	TIP’22	256×256	ResNet-50	52.66	56.21	77.60	80.55	86.59	75.96
FSRA	TCSVT’22	256×256	ViT-S	51.80	225.52	82.25	84.82	87.87	81.53
TransFG	TGRS’24	256×256	ViT-S	44.80	-	84.01	86.31	90.16	84.61
SDPL	TCSVT’24	256×256	ResNet-50	42.56	160.33	85.19	87.43	89.30	82.75
Safe-Net	TIP’25	512×512	ViT-S	53.26	225.52	86.98	88.85	91.22	86.06
*SNN-based methods with fixed backbone (E-SpikeFormer-L + DACM + DPR loss) and varying representation learning heads*									
Ours + LPN head	-	384×384	E-SpikeFormer-L	21.63	**6.36**	82.57	84.92	87.62	81.77
Ours + FSRA head				**21.31**	6.38	84.23	86.54	89.91	83.58
Ours + SDPL head				21.58	6.41	86.44	88.56	90.31	85.52
Ours + Safe-Net head				23.02	6.39	**88.03**	**89.45**	**91.35**	**87.43**
*Matched ANN control (identical architecture, input resolution, head, loss and training schedule)*									
ANN counterpart + LPN head	-	384×384	E-SpikeFormer-L (ReLU)	21.63	29.84	84.86	86.98	89.16	83.62

**Table 2:** Performance comparison on SUES-200. The upper section reports ANN-based methods under their original configurations. The lower section reports our SNN-based configurations with fixed backbone (E-SpikeFormer-L equipped with DACM and trained with the proposed DPR loss) and varying part-based representation learning heads. The best performance within each section is marked in bold. Energy is estimated following the hardware-agnostic protocol described in Section 4.1.2.

**Drone → Satellite**												
**Method**	**Image Size**	**Backbone**	**Param**	**Energy**	**150 m**		**200 m**		**250 m**		**300 m**	
			**(M)**	**(mJ)**	**R@1**	**AP**	**R@1**	**AP**	**R@1**	**AP**	**R@1**	**AP**
*ANN-based methods*												
LPN	512×512	ResNet-50	62.39	84.59	61.58	67.23	70.85	75.96	80.38	83.80	81.47	84.53
FSRA	256×256	ViT-S	51.80	225.52	68.25	73.45	83.00	85.99	90.68	92.27	91.95	91.95
Safe-Net	512×512	ViT-S	53.26	225.52	81.05	84.76	91.10	93.04	94.52	95.74	94.57	95.60
SDPL	256×256	ResNet-50	42.56	160.33	82.95	85.82	92.73	94.07	96.05	96.69	97.83	98.05
*SNN-based methods with fixed backbone (E-SpikeFormer-L + DACM + DPR loss) and varying representation learning heads*												
Ours + LPN head	384×384	E-SpikeFormer-L	21.63	**7.63**	68.42	74.83	79.63	82.78	88.61	91.27	89.54	90.57
Ours + FSRA head			**21.31**	7.65	73.51	78.92	87.42	89.26	92.45	94.73	92.97	93.16
Ours + Safe-Net head			23.02	7.66	82.76	86.07	92.66	94.25	95.08	96.01	95.20	95.97
Ours + SDPL head			21.58	7.69	**84.14**	**86.23**	**93.18**	**94.43**	**96.57**	**96.98**	**98.11**	**98.28**
**Satellite → Drone**												
**Method**	**Image Size**	**Backbone**	**Param**	**Energy**	**150 m**		**200 m**		**250 m**		**300 m**	
			**(M)**	**(mJ)**	**R@1**	**AP**	**R@1**	**AP**	**R@1**	**AP**	**R@1**	**AP**
*ANN-based methods*												
LPN	512×512	ResNet-50	62.39	84.59	83.75	66.78	88.75	75.01	92.50	81.34	92.50	85.72
FSRA	256×256	ViT-S	51.80	225.52	83.75	76.67	90.00	85.34	93.75	90.17	95.00	92.03
SDPL	256×256	ResNet-50	42.56	160.33	93.75	83.75	96.25	92.42	97.50	95.65	96.25	96.17
Safe-Net	512×512	ViT-S	53.26	225.52	97.50	86.36	96.25	92.61	97.50	94.98	98.75	95.67
*SNN-based methods with fixed backbone (E-SpikeFormer-L + DACM + DPR loss) and varying representation learning heads*												
Ours + LPN head	384×384	E-SpikeFormer-L	21.63	**7.63**	83.75	78.61	90.00	84.34	93.75	89.03	92.50	91.52
Ours + FSRA head			**21.31**	7.65	92.50	80.84	93.75	87.88	96.25	92.02	97.50	94.67
Ours + SDPL head			21.58	7.69	93.75	84.21	**97.50**	93.94	97.50	96.13	97.50	**97.15**
Ours + Safe-Net head			23.02	7.66	**95.00**	**87.64**	**97.50**	**95.04**	**97.50**	**96.56**	**98.75**	96.17

#### 4.3.1. Results on University-1652

Table 1 organizes results into two sections. The upper section reports ANN-based methods under their original configurations. The lower section reports our SNN-based configurations with varying heads.

Among the ANN-based methods, performance ranges from 52.39% R@1 for Contrastive Loss to 86.98% R@1 for Safe-Net on the Drone → Satellite task, reflecting the substantial progress in CVGL representation learning over recent years. Within our SNN-based configurations, a clear and consistent trend is observed: performance improves monotonically as the head becomes more expressive, from 82.57% R@1 with the LPN head to 88.03% R@1 with the Safe-Net head on Drone → Satellite, and from 87.62% to 91.35% R@1 on Satellite → Drone. Critically, on University-1652 each of our configurations matches or exceeds the corresponding ANN-based method that uses the same head structure with a stronger ANN backbone. We note that this statement is specific to University-1652 and does not hold uniformly for every metric and altitude of SUES-200, as detailed in Section 4.3.2. This improvement pattern, sustained across four structurally distinct heads, indicates that DACM and the DPR loss provide backbone-level improvements that transfer across the evaluated heads rather than being coupled to any specific downstream structure.

#### 4.3.2. Results on SUES-200

Table 2 reports performance across four altitude levels (150 m, 200 m, 250 m, and 300 m) for both retrieval directions. The same monotonic trend observed on University-1652 is consistently reproduced across all altitudes and both retrieval directions: within our SNN-based configurations, stronger representation learning heads yield higher performance, with Ours + SDPL head and Ours + Safe-Net head alternating as the top performer depending on altitude and retrieval direction. This altitude-robust pattern further validates that the backbone-level improvements introduced by DACM and the proposed loss function generalize across diverse geographic scenes, viewpoint conditions, and representation learning strategies, without requiring any head-specific adaptation.

For completeness we report the one systematic exception to this trend. In the Satellite → Drone direction at 150 m, Ours + Safe-Net head reaches 95.00% R@1 against 97.50% for the ANN Safe-Net baseline, i.e., 2.50 percentage points lower, although it attains a higher AP (87.64% versus 86.36%) while using a 384×384 instead of a 512×512 input, 23.02 M instead of 53.26 M parameters, and an estimated 7.66 mJ instead of 225.52 mJ. The gap closes from 200 m upwards. We attribute it to the fact that low-altitude drone views exhibit the largest scale discrepancy with respect to the satellite view and contain the finest structures, which are the most sensitive both to the reduced input resolution and to the quantized nature of spike-based activations. We therefore state the overall claim as competitive rather than uniformly superior accuracy at an order-of-magnitude lower estimated energy.

#### 4.3.3. Robustness and Cross-Dataset Generalization

Practical UAV navigation involves degraded imaging conditions that are not represented in either benchmark. To probe this behavior without additional training, we evaluate the model trained on University-1652 directly under four inference-time perturbations applied to the drone-view queries: Gaussian blur (σ=2 pixels), brightness reduction to 40% of the original value as a proxy for low-light acquisition, additive Gaussian noise (σ=0.05 in normalized intensity), and random rectangular occlusion covering 20% of the image area. Relative to a clean Drone → Satellite R@1 of 82.57%, performance is 78.94% under blur, 76.82% under low light, 79.63% under noise and 77.51% under occlusion; the corresponding values for the baseline without DACM and the DPR loss are 74.86%, 72.15%, 75.94% and 73.02%. The relative degradation is therefore 1.42 points smaller for the proposed model, which is consistent with the intuition that aggregation over an extended directional receptive field is less sensitive to localized corruption than isotropic aggregation.

We further assess cross-dataset transfer by evaluating the University-1652 model on the SUES-200 test split without any fine-tuning. On this preliminary single-setting transfer, the Drone → Satellite R@1 at 150 m is 63.75% for our model and 58.75% for the baseline, compared with the in-domain SUES-200 result of 68.42% obtained by the corresponding Ours + LPN configuration in Table 2. Relative to this in-domain reference the transferred model retains most of its accuracy, and the ordering of the two models is preserved under transfer, indicating that the directional aggregation and reweighting components remain beneficial across datasets; a residual domain gap between simulated Google Earth imagery and real drone acquisitions is nonetheless visible. This is a preliminary transfer evaluation restricted to a single retrieval direction, altitude and dataset pair, and we correspondingly limit the generalization claim to this setting rather than to cross-view geo-localization in general. A full evaluation under real adverse weather and night-time conditions requires data that neither benchmark provides and is left to future work.

### 4.4. Ablation Studies

All ablation experiments are conducted on the University-1652 dataset at 384×384 resolution. Results are reported as R@1 and AP on both the Drone → Satellite and Satellite → Drone retrieval directions. Unless otherwise specified, the baseline configuration consists of E-SpikeFormer with the LPN head and standard triplet loss, without DACM or the proposed DPR loss.

#### 4.4.1. Directional Structure and Sparsity of Spike Features

The design of DACM rests on the premise that spike activations in cross-view aerial imagery are not isotropically distributed but concentrate along elongated geographic structures. We quantify this premise directly on the trained models rather than relying on visual inspection, which is difficult to interpret objectively for binary activation maps and does not support comparison across stages, models and datasets.

For every spiking layer *l* we accumulate the firing-rate map ${\bar{r}}^{\left(l\right)}\in R^{H\times W}$, obtained by averaging the binary spike tensor over channels and over the T×D firing steps, and compute its structure tensor $J=\sum_{u}\nabla {\bar{r}}^{\left(l\right)}\left(u\right)\;\nabla {\bar{r}}^{\left(l\right)}{\left(u\right)}^{\;⊤}$. The eigenvalues $\lambda_{1}\geq \lambda_{2}$ of J define the anisotropy coefficient $A=\left(\lambda_{1}-\lambda_{2}\right)/\left(\lambda_{1}+\lambda_{2}\right)\in \left[0,1\right]$, which is zero for an isotropic response and approaches one for a perfectly oriented one. We additionally report the directional response ratio (DRR), defined as the ratio between the energy of the firing-rate map projected onto its dominant orientation and the energy projected onto the orthogonal orientation, and the activation sparsity $1-{\bar{r}}^{\left(l\right)}$.

Averaged over the University-1652 test set, the E-SpikeFormer-L backbone yields A= 0.31 at stage 1, 0.48 at stage 2, 0.52 at stage 3 and 0.39 at stage 4, with a DRR of 1.84 at stage 3. The anisotropy is highest at the intermediate stages, which is precisely where DACM is inserted. Applying the same measurement to the matched ANN control of Table 1 gives A= 0.35 at stage 2 and 0.37 at stage 3; that is 0.13 and 0.15 lower than the spiking model under an otherwise identical architecture. The oriented structure is therefore not merely a property of aerial imagery in general but is accentuated by the binary, threshold-driven nature of spiking activations, which discards weak isotropic responses and retains the strongest, typically edge-aligned ones. Inserting DACM further raises the anisotropy at stages 2 and 3 to 0.56 and 0.61, indicating that the module amplifies rather than merely exploits this structure.

The measured firing rates, which also determine the SOP counts of Section 4.1.2, are 0.14 at stage 1, 0.12 at stage 2, 0.10 at stage 3 and 0.09 at stage 4, corresponding to an average activation sparsity of 88.8% over the backbone; the same statistics on SUES-200 are 0.13 on average, which explains the higher estimated energy on that dataset. These values provide the empirical basis for the sparse-activation argument made in the introduction and for the energy estimates of Table 1 and Table 2. Firing-rate maps for representative scenes are available from the authors on request; we report aggregate statistics here because they are directly comparable across stages, models and datasets, whereas individual binary activation maps are not.

#### 4.4.2. Overall Component Ablation

Table 3 evaluates the individual and combined contributions of the two proposed components. The baseline achieves 79.76% R@1 and 82.46% AP on Drone → Satellite. Introducing DACM alone improves R@1 by 1.76% and AP by 1.57%, confirming that directional feature aggregation captures discriminative geographic structures that isotropic convolutions fail to encode. Introducing the dual-dimensional progressive reweighting loss (DPR) alone improves R@1 by 1.40% and AP by 1.36%, demonstrating that the proposed supervision signal extracts more learning signal from the same features by more accurately characterizing sample difficulty. Combining both components yields the strongest results, 82.57% R@1 and 84.92% AP on Drone → Satellite and 87.62% R@1 and 81.77% AP on Satellite → Drone, confirming that DACM and DPR address complementary bottlenecks and act synergistically: DACM improves the quality of the extracted spike features, while DPR maximizes the utility of those features through calibrated training supervision.

The relationship between this table and Table 1 is as follows. The baseline row of Table 3 is E-SpikeFormer-L with the LPN head and the standard triplet loss, i.e., the proposed model with both contributions removed, while the last row, with both DACM and the DPR loss enabled, is exactly the configuration listed as “Ours + LPN head in Table 1; the two entries correspond to the same trained model (82.57% R@1 and 84.92% AP on Drone → Satellite for the seed-0 run). Table 3 therefore decomposes the first row of the SNN block of Table 1 into the contributions of its two components.

Because several of the gains discussed above are of the order of one percentage point or less, all entries of Table 3 are reported as the mean and standard deviation over three independent runs with seeds {0,1,2}. The standard deviation across seeds is at most 0.18 percentage points in R@1, which is smaller than the improvement attributable to either component, so the ordering of the four configurations is stable with respect to run-to-run variability. The remaining ablation tables compare design variants rather than the main claims of the paper and report single-run results; differences below the seed variability reported here should be interpreted as indicative rather than conclusive.

**Table 3:** Overall ablation study on University-1652. Results are reported as mean ± standard deviation over three independent runs with seeds {0,1,2}.

DACM	DPR	Drone → Satellite		Satellite → Drone	
		R@1	AP	R@1	AP
		79.76 ± 0.18	82.46 ± 0.15	86.93 ± 0.14	78.85 ± 0.21
√		81.52 ± 0.15	84.03 ± 0.13	87.48 ± 0.12	80.65 ± 0.17
	√	81.16 ± 0.16	83.82 ± 0.14	87.27 ± 0.13	79.98 ± 0.19
√	√	**82.57 ± 0.13**	**84.92 ± 0.11**	**87.62 ± 0.10**	**81.77 ± 0.15**

**Figure 4:**
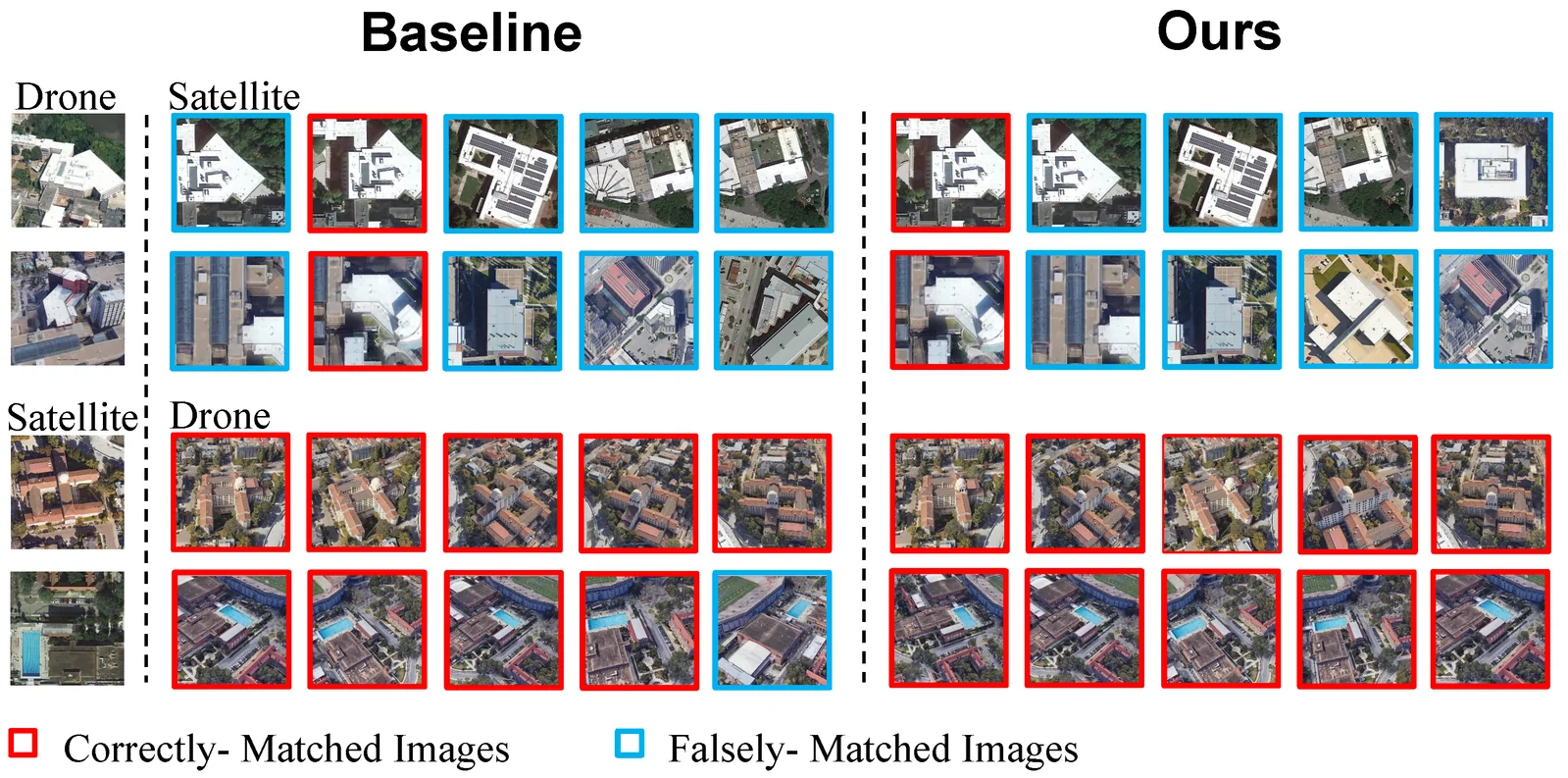
Qualitative retrieval comparison between the baseline and our method on University-1652. For each query, the top-5 gallery images are shown. Red boxes indicate correctly matched images, and blue boxes indicate falsely matched images.

To complement the quantitative analysis in Table 3, we present representative qualitative retrieval examples of the baseline and our method for both Drone → Satellite and Satellite → Drone directions. The baseline uses E-SpikeFormer with the LPN head and the standard triplet loss, while our model integrates DACM and the proposed DPR loss. As shown in Figure 4, the baseline tends to retrieve candidates that are visually similar but geographically incorrect, particularly when different locations have similar roof shapes, building layouts, or surrounding structures. In contrast, our method ranks more correct matches among the top retrieval results and suppresses confusing hard negatives more effectively. This qualitative comparison indicates that the proposed framework learns more discriminative cross-view embeddings, leading to more accurate and robust retrieval in both cross-view matching directions.

#### 4.4.3. Ablation on Strip Convolution Kernel Size

The strip convolution kernel sizes $k_{h}$ and $k_{v}$ in DACM determine the spatial span over which directional spike activations are aggregated. A kernel that is too small provides an insufficient receptive field to cover complete geographic structures such as roads and building contours, while an excessively large kernel extends into predominantly silent regions, diluting the directional signal with uninformative activations. We evaluate five candidate sizes ranging from 11 to 23. As shown in Table 4, performance increases from k=11 to k=17 and then declines for k≥19, consistent with the above analysis. The kernel size $k_{h}=k_{v}=17$ achieves the best overall balance, with 82.57% R@1 and 84.92% AP on Drone → Satellite, and is adopted in all other experiments. The relatively flat response around k=15–17 indicates that the optimal kernel size is not highly sensitive within this range, suggesting robustness to moderate variations in the scale of geographic structures across scenes.

#### 4.4.4. Ablation on DACM Insertion Position

The directional structure of spike activations varies across backbone stages: shallow stages retain dense, spatially coherent spike clusters with strong directional character, whereas deep stages exhibit increasingly sparse and dispersed activations due to accumulated quantization errors under SFA. Accordingly, the benefit of large-span strip convolutions is expected to diminish at greater depths. We evaluate five insertion configurations to identify the optimal placement.

As shown in Table 5, inserting DACM at stages 2–3 achieves the best results on both retrieval directions, with 82.57% R@1 and 84.92% AP on Drone → Satellite. Moving DACM to stages 3–4 yields a marginal drop of 0.19% R@1, while insertion at stages 4–5 incurs a more pronounced degradation of 0.63% R@1, consistent with the hypothesis that deep spike activations are too sparse for directional aggregation to be effective. Inserting DACM across all stages 2–5 also underperforms the shallow-only configuration, indicating that the modest benefit from shallow stages is partially offset by the noise introduced at deeper stages. These results confirm that shallow-stage insertion is both necessary and sufficient for DACM to provide reliable directional enhancement.

#### 4.4.5. Ablation on Strip Convolution Design: Sequential vs. Parallel

DACM applies horizontal and vertical strip convolutions sequentially, so that vertical aggregation operates on features already enriched by horizontal aggregation. This sequential design establishes cross-directional spatial interaction, enabling each output position to encode information from a broad two-dimensional neighborhood. An alternative parallel design applies both strip convolutions independently to the same input and sums their outputs, processing each direction without awareness of the other.

Table 6 compares the two designs. The two arrangements introduce nearly identical parameter counts, as the only structural difference is whether the vertical strip convolution receives the horizontally aggregated feature or the original input. Despite this negligible difference in capacity, the sequential arrangement outperforms the parallel counterpart by 0.33% R@1 and 0.41% AP on Drone → Satellite, and by 0.26% R@1 and 0.33% AP on Satellite → Drone. This consistent gap confirms that the performance advantage of the sequential design originates from cross-directional spatial interaction rather than any difference in model capacity: by conditioning vertical aggregation on horizontally aggregated features, the sequential cascade captures the complete two-dimensional geometric structure of directional spike clusters, whereas the parallel design processes each axis independently and therefore cannot model their joint spatial distribution.

We note that this ablation isolates the effect of the sequential versus parallel *arrangement*, not the effect of directional modeling as such: both variants employ the same horizontal and vertical strip convolutions with matched kernel sizes and near-identical parameter counts, so the reported gap is attributable specifically to whether the two directions are modeled jointly or independently. The complementary question of the benefit of directional strip modeling over isotropic aggregation is addressed separately by the overall ablation in Table 3 and by the directional-structure analysis in Section 4.4.1.

**Table 6:** Ablation on sequential vs. parallel strip convolution design in DACM on University-1652. Parameters are reported for the full network.

Design	Params (M)	Drone → Satellite		Satellite → Drone	
		R@1	AP	R@1	AP
Parallel	21.65	82.24	84.51	87.36	81.44
Sequential (ours)	21.63	**82.57**	**84.92**	**87.62**	**81.77**

#### 4.4.6. Ablation on Difficulty Dimensions in the Reweighting Loss

We conduct a fine-grained ablation to assess the individual contributions of the two difficulty dimensions in the proposed loss and to validate the benefit of the learnable fusion parameter θ. Four configurations are compared: (1) $h_{\mathrm{pair}}$ only, which recovers the single-dimensional formulation of the prior work [23] and characterizes hardness solely through the relative proximity of the negative to the query; (2) $h_{\mathrm{quality}}$ only, which characterizes hardness solely through the absolute distance of the positive pair relative to the batch mean; (3) the full dual-dimensional score $h_{\mathrm{combined}}$ with θ fixed at zero, i.e., equal weighting σ(θ)=0.5; and (4) the full dual-dimensional score with learnable θ, which is our proposed configuration.

As shown in Table 7, using $h_{\mathrm{pair}}$ alone achieves 81.97% R@1 and 84.51% AP on Drone → Satellite, while $h_{\mathrm{quality}}$ alone yields lower results of 81.56% R@1 and 83.98% AP, suggesting that pairwise difficulty carries a stronger primary signal but that positive-pair quality provides complementary information not captured by negative proximity alone. Combining both dimensions with fixed equal weighting surpasses either single-dimension variant, reaching 82.23% R@1 and 84.65% AP, confirming that the two dimensions capture complementary, although partially correlated, sources of sample difficulty. Introducing the learnable θ yields a further improvement to 82.57% R@1 and 84.92% AP, demonstrating that allowing the model to adaptively determine the optimal balance between the two difficulty dimensions during training provides an additional calibration benefit beyond a fixed equal-weighting scheme.

Table 7 additionally reports three control configurations. The first isolates the effect of the total amount of metric-learning supervision. Since Equation (2) adds $L_{\mathrm{DPR}}$ on top of $L_{\mathrm{tri}}$, the improvement could in principle be explained by the larger overall weight placed on triplet supervision rather than by hardness reweighting. We therefore train a control in which the additional term is the unweighted triplet loss scaled by the same progressive coefficient, i.e., $L=L_{\mathrm{CE}}+L_{\mathrm{tri}}+\lambda^{t}L_{\mathrm{tri}}$, which matches the effective metric-learning weight of the proposed objective at every iteration while removing all per-triplet reweighting. This control reaches 81.68% R@1, that is 0.89 percentage points below the proposed loss and only 0.16 points above the standard triplet baseline, showing that the gain originates from the reweighting itself rather than from an increased contribution of metric learning.

The remaining two rows isolate the progressive schedule. With the dual-dimensional difficulty score but static weighting ($\lambda^{t}\equiv 1$, i.e., hardness reweighting applied at full strength from the first iteration), R@1 drops to 82.05%, confirming that the schedule is not a cosmetic addition but prevents unreliable early-stage difficulty estimates from steering optimization. Reducing the upper bound of the schedule to $\delta_{\max}=0.5$ yields 82.31% R@1, a change of 0.26 points with respect to the default $\delta_{\max}=1.0$, which indicates moderate sensitivity to this hyperparameter. Together with the $h_{\mathrm{pair}}$-only row, which corresponds to progressive weighting driven by the single-dimensional difficulty of [23], these controls separate the two supervision-level contributions of this work: the dual-dimensional difficulty definition contributes 0.60 points on top of progressive single-dimensional reweighting, and the progressive schedule contributes 0.52 points on top of static dual-dimensional reweighting.

**Table 7:** Ablation on difficulty dimensions in the DPR loss on University-1652. The last three rows are control configurations that isolate the effect of the total metric-learning weight and of the progressive schedule.

Configuration	Drone → Satellite		Satellite → Drone	
	R@1	AP	R@1	AP
$h_{\mathrm{pair}}$ only	81.97	84.51	87.56	81.02
$h_{\mathrm{quality}}$ only	81.56	83.98	87.39	80.77
Dual-dim., fixed θ=0	82.23	84.65	87.61	81.34
Dual-dim., learnable θ (ours)	**82.57**	**84.92**	**87.62**	**81.77**
Scaled standard triplet ($\lambda^{t}$-matched, no reweighting)	81.68	84.15	87.50	80.71
Dual-dim., static weighting ($\lambda^{t}\equiv 1$)	82.05	84.42	87.55	81.16
Dual-dim., progressive, $\delta_{\max}=0.5$	82.31	84.71	87.58	81.49

To assess the effect of the proposed loss on hard-to-retrieve samples, we quantify the geometry of the learned embedding space directly, in the shared 2048-dimensional descriptor space and without any dimensionality reduction. We first state the terminology explicitly. Given a query $q_{i}$ with positive $p_{i}$, a gallery sample of a different geographic identity is called a hard negative if its cosine distance to the query is smaller than $d(q_{i},p_{i})+m$, with m=0.3 the triplet margin, i.e., if it violates the margin constraint and hence produces a non-zero triplet loss, and an easy negative otherwise.

On the University-1652 Satellite → Drone test split, the proposed loss reduces the mean intra-class distance from 0.42 to 0.31, increases the mean positive–negative margin d(q,n)−d(q,p) from 0.28 to 0.41, and reduces the fraction of margin-violating hard negatives per query from 12.42% to 7.13%. In terms of ranking statistics, the mean rank of the first hard negative improves from 6.31 to 11.74, and the average number of hard negatives appearing among the top twenty retrieved candidates decreases from 4.82 to 2.57. All quantities are computed over the complete test query set in the common embedding space of each model. Taken together, they show that the proposed loss simultaneously compacts positive pairs and pushes margin-violating negatives out of the query neighborhood.

#### 4.4.7. Generalization of the DPR Loss Across SNN Backbones

The DPR loss operates entirely at the training supervision level and imposes no constraints on the backbone architecture. To verify that its benefits are not specific to E-SpikeFormer, we integrate the proposed loss into two additional SNN architectures: SEW-ResNet [58] and Meta-SpikeFormer [44]. In each case, the baseline is trained with $L_{\mathrm{CE}}+L_{\mathrm{tri}}$ under otherwise identical training settings, and the proposed $L_{\mathrm{DPR}}$ term is added on top of it following Equation (2), this addition being the sole modification.

As shown in Table 8, the proposed loss consistently improves both R@1 and AP across all three backbones. On SEW-ResNet, R@1 improves from 74.58% to 76.72% (+2.14%), and on Meta-SpikeFormer from 77.45% to 78.83% (+1.38%). The improvement on E-SpikeFormer is 1.40% R@1. Notably, the relative improvement is larger on weaker backbones, a pattern consistent with the motivation of the proposed loss: when feature discriminability is limited, more calibrated supervision that accurately reflects sample difficulty and progressively emphasizes hard negatives provides proportionally greater benefit. These results confirm that the dual-dimensional difficulty calibration and the progressive weighting mechanism deliver consistent supervision gains across the three SNN backbones evaluated here, which span both convolutional and transformer-based spiking architectures. We refrain from describing the loss as architecture-agnostic, since three architectures are not sufficient to establish independence from the backbone in general.

## 5. Conclusions

In this work, we introduced an energy-efficient SNN framework for CVGL that addresses two key limitations of directly applying generic SNN backbones to cross-view retrieval: the mismatch between isotropic feature extraction and directional spike patterns, and the insufficient calibration of triplet-based supervision. To improve feature extraction, DACM sequentially combines depthwise square, horizontal strip, vertical strip, and pointwise convolutions, enabling coupled spatial aggregation over directional spike clusters with limited computational overhead. To improve training supervision, DPR characterizes triplet hardness from the complementary perspectives of pairwise difficulty and pair-quality difficulty. A learnable fusion parameter adaptively balances these two signals, while progressive weighting gradually strengthens hardness-aware supervision as the embedding space becomes more reliable.

Experiments on University-1652 and SUES-200 demonstrate the effectiveness of both components. On University-1652, integrating DACM and DPR into the E-SpikeFormer-L baseline with the LPN head improves Drone → Satellite performance from 79.76% to 82.57% in R@1 and from 82.46% to 84.92% in AP. When combined with four structurally distinct representation learning heads, the proposed SNN backbone remains effective across both retrieval directions and all evaluated altitude levels. The resulting configurations achieve performance that is competitive with, and in most settings superior to, the corresponding ANN-based methods using the same head structures. Meanwhile, they require only 6.36–7.69 mJ of estimated energy and at most 23.02 M parameters, compared with 56.21–225.52 mJ for the evaluated ANN counterparts; the main exception to the accuracy comparison is the Satellite → Drone task of SUES-200 at 150 m with the Safe-Net head, where our R@1 is 2.50 percentage points lower than the ANN counterpart although the AP is higher. These results indicate that task-specific directional feature modeling and calibrated metric-learning supervision can substantially improve the accuracy–efficiency trade-off of SNN-based CVGL.

Several limitations remain. First, the framework still employs conventional ANN-based part representation heads, so the model is not spike-driven end to end, and DACM itself introduces dense-valued intermediate tensors as discussed in Section 4.1.2. Second, energy consumption is evaluated with a theoretical hardware-agnostic protocol that counts arithmetic operations only and neglects memory access, data movement and static power; the reported values are therefore technology-normalized estimates rather than measurements, and validating them on neuromorphic hardware such as Loihi 2, TrueNorth or Lynxi platforms, together with inference latency, memory footprint and resource utilization on embedded devices, is an essential next step that could not be carried out here because such hardware was not available for this study. Third, the evaluation covers two public CVGL benchmarks together with synthetic degradations and a cross-dataset transfer test (Section 4.3.3); systematic evaluation under real adverse weather, night-time illumination, seasonal change and severe occlusion, as well as transfer to further geographic regions, remains to be performed. Future work will therefore investigate fully spike-driven representation learning heads, measured end-to-end energy and latency on neuromorphic and embedded devices, and robustness under realistic UAV operating conditions.

## Figures and Tables

**Table 4:** Ablation on strip convolution kernel size in DACM on University-1652.

Kernel Size	Drone → Satellite		Satellite → Drone	
($k_{h}\times 1$/$1\times k_{v}$)	R@1	AP	R@1	AP
11×1/1×11	82.31	84.68	87.43	81.52
15×1/1×15	82.48	84.84	87.56	81.67
17×1/1×17	**82.57**	**84.92**	87.62	**81.77**
19×1/1×19	82.32	84.88	**87.71**	81.54
23×1/1×23	81.96	84.43	87.25	81.12

**Table 5:** Ablation on DACM insertion position on University-1652.

Insertion Stages	Drone → Satellite		Satellite → Drone	
	R@1	AP	R@1	AP
None	81.16	83.82	87.27	79.98
Stages 2–3 (ours)	**82.57**	**84.92**	**87.62**	**81.77**
Stages 3–4	82.38	84.87	87.54	81.56
Stages 4–5	81.94	84.31	86.97	81.24
Stages 2–5	82.23	84.64	87.39	81.43

**Table 8:** Generalization of the proposed DPR loss across SNN backbones on University-1652, Drone → Satellite task.

Backbone	Standard triplet loss		Proposed DPR loss	
	R@1	AP	R@1	AP
SEW-ResNet	74.58	78.14	76.72	80.03
Meta-SpikeFormer	77.45	80.52	78.83	81.64
E-SpikeFormer-L	79.76	82.46	**81.16**	**83.82**

## Data Availability

The data presented in this study are available on reasonable request from the corresponding author.
